# Triphasic spike-timing-dependent plasticity organizes networks to produce robust sequences of neural activity

**DOI:** 10.3389/fncom.2012.00088

**Published:** 2012-11-12

**Authors:** Amelia Waddington, Peter A. Appleby, Marc De Kamps, Netta Cohen

**Affiliations:** ^1^School of Computing, University of LeedsLeeds, UK; ^2^Institute of Systems and Membrane Biology, University of LeedsLeeds, UK

**Keywords:** activity dependent plasticity, computational model, microcircuits, network development, random walk, songbird, synfire chains, zebra finch

## Abstract

Synfire chains have long been proposed to generate precisely timed sequences of neural activity. Such activity has been linked to numerous neural functions including sensory encoding, cognitive and motor responses. In particular, it has been argued that synfire chains underlie the precise spatiotemporal firing patterns that control song production in a variety of songbirds. Previous studies have suggested that the development of synfire chains requires either initial sparse connectivity or strong topological constraints, in addition to any synaptic learning rules. Here, we show that this necessity can be removed by using a previously reported but hitherto unconsidered spike-timing-dependent plasticity (STDP) rule and activity-dependent excitability. Under this rule the network develops stable synfire chains that possess a non-trivial, scalable multi-layer structure, in which relative layer sizes appear to follow a universal function. Using computational modeling and a coarse grained random walk model, we demonstrate the role of the STDP rule in growing, molding and stabilizing the chain, and link model parameters to the resulting structure.

## 1. Introduction

The ability of the brain to process information quickly, reliably and reproducibly is an important challenge for neuroscience. One solution that the brain appears to use is precisely timed and sequential activity patterns. First conjectured in order to account for such sequential patterns (Abeles, 1982, 1991), synfire chains are effectively feed-forward structures composed of multiple layers in which the activity flows from the input, sequentially through the layers, with each repetition of the input producing the same precise firing pattern.

Accumulating evidence from electrophysiological recordings demonstrates that such precise sequences of neural activity occur regularly *in vivo*. For example, it has been shown that precise chains of activity can determine visual input (Ayzenshtat et al., 2010) in monkeys and predict their reaching (Abeles et al., 1993), opening a puzzle box (Prut et al., 1998), moving a joystick (Hatsopoulos et al., 2003), and drawing (Shmiel et al., 2006). In rats, neural sequences can be used to predict the behavioral response to auditory cues (Villa et al., 1999). There are also reports that the spontaneous activity displayed by systems at rest contain repeating sequential patterns (Mao et al., 2001; Luczak et al., 2007). Indeed, the latter reports suggest that such patterns could be generated by the underlying circuit even in the absence of structured input.

Although spatiotemporal activity patterns have been found in a range of mammalian preparations, by far the most concrete example of precisely timed neural sequences with a direct link to a specific behavior can be found in a variety of song birds. The zebra finch is a particularly interesting song bird as it, like humans, learns stereotyped vocal behavior, usually from its parents. In adult birds, song production is primarily undertaken by the feed-forward song motor pathway; higher vocal center (HVC) neurons control the firing of neurons in the robust nucleus of the arcopallium (RA), which control the vocal output through the telencephalon (Mooney, 2009; Ölveczky and Gardner, 2011). The evidence suggests that HVC orchestrates song production (Mooney, 1992; Brainard and Doupe, 2000; Long and Fee, 2008) and the sequential and precisely timed activity patterns found here are exactly correlated to song vocalization on a sub-millisecond timescale (Hahnloser et al., 2002). One hypothesis is that synfire chains are responsible for these firing patterns (Long et al., 2010) and recent computational models based on collections of synfire chains and feedback inhibition have successfully reproduced HVC behavior in normal (Jin, 2009) and deafened birds (Hanuschkin et al., 2011).

The conjectured importance of synfire chains has inspired a large body of computational work, which has demonstrated the capacity of synfire chains to generate precise sequences of spikes (Abeles, 1982, 1991; Diesmann et al., 1999). Extensions include investigations into the role of such structures in working memory (Kitano et al., 2003; Aviel et al., 2005; Ishibashi et al., 2006), within balanced networks (Gewaltig et al., 2001; Aviel et al., 2003; Tetzlaff et al., 2004; Kumar et al., 2008; Trengove et al., 2012), in the presence of noise (van Rossum et al., 2002; Ikeda, 2003), and with inhibitory modulation (Shinozaki et al., 2010).

In the context of songbirds, strictly feed-forward synfire chains are thought to encode the *syllables* that make up the song (Jin, 2009; Fiete et al., 2010; Hanuschkin et al., 2011). In particular, each syllable consists of a sequence of *notes*, that may be encoded by the layers in the chain. Accordingly, the sequences of notes within the syllable are very precisely timed over a syllable with of *O*(100) ms overall duration (Brainard and Doupe, 2002; Glaze and Troyer, 2006). The sequences of syllables that then form motifs and songs involve additional syntax, that likely require the formation of loops in the chain, synfire braids or other compositional structures (Jin, 2009; Hanuschkin et al., 2011).

**Figure 1:**
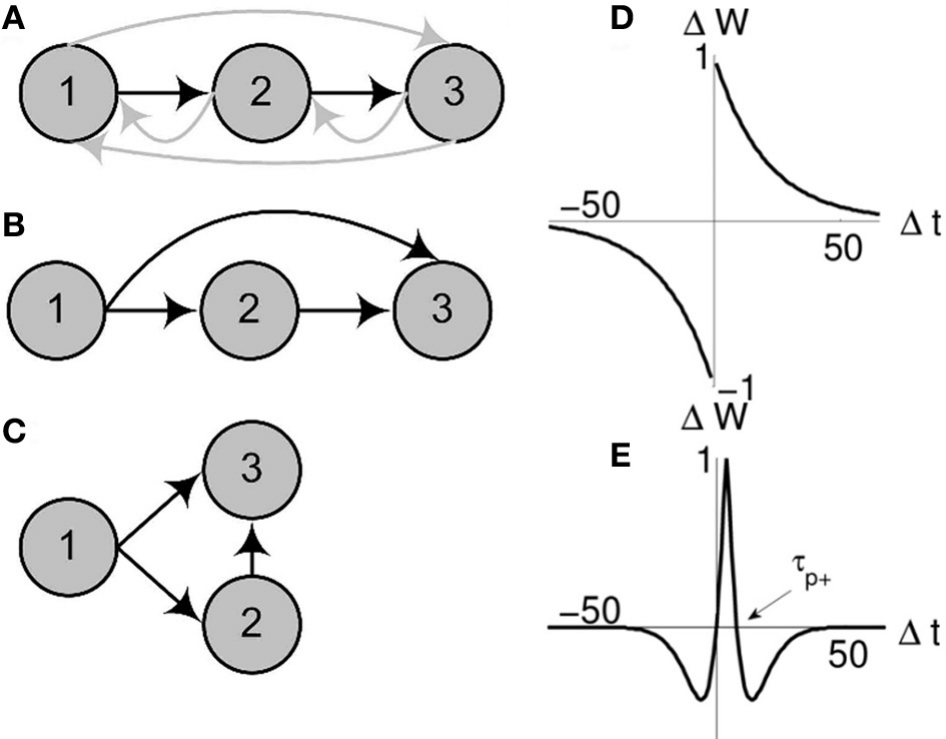
**Schematic of network collapse.** Evolution of an existing chain under classical and triphasic STDP rules. For an initial network configuration **(A)** evolving under classical STDP **(D)**, the initially sub-threshold connection from neuron 1 to neuron 3 will potentiate **(B)**. When sufficiently strong (as strong as the initial suprathreshold connection from neuron 1 to neuron 2), spikes in neuron 1 will propagate in parallel to neurons 1 and 3, causing both neurons to spike synchronously. At this point layers 2 and 3 of the chain are said to have collapsed **(C)**. If the chain **(A)** develops under triphasic STDP **(E)**, the chain structure is stable (all far-forward or backward connections are depressed).

Naturally, the question of synfire chain development is also of much interest. Early work on the development of synfire chains (Bienenstock, 1991, 1992) utilized Hebbian plasticity. More recently, the discovery of spike-timing-dependent plasticity (STDP) provided a new impetus to model the *ab initio* development of synfire chains. Previous computational studies on developing synfire chains have focused on a specific STDP rule that was first reported by Bi and Poo (1998) for rat hippocampal cell cultures (Figure 1D). In this temporally asymmetrical function, apparently causal firing patterns lead to the potentiation of the corresponding synapse whereas apparently anti-causal firing patterns lead to its depression (Abbott et al., 2000; Bi and Poo, 2001; Caporale and Dan, 2008). In part because of its intuitive appeal, this general form of the STDP rule has since become the *classical* STDP rule in computational neuroscience, and henceforth we refer to it as such^1^.

The classical STDP rule is particularly appealing in the context of synfire chain development. Specifically, the repeated potentiation of forward projections and the depression of backward projections appear conducive to the development of chains. Indeed, many authors (Doursat and Bienenstock, 2006; Jun and Jin, 2007; Masuda and Kori, 2007; Hosaka et al., 2008; Iglesias and Villa, 2008; Fiete et al., 2010) have successfully demonstrated development of synfire chains using variants of this classical STDP rule. Note however, that in all these studies, the STDP rule was complemented by additional mechanisms that served to limit the number of synaptic partners a neuron can have. If projections from the input are consistently potentiated, these efferent (forward) projections will be limited only by the potential connectivity of the network. For a fully connected network, inputs will project directly onto the entire network. To prevent this, topological constraints are imposed.

There are different ways to impose such topological constraints. Perhaps the most straightforward approach is to limit the initial connectivity of the network (Masuda and Kori, 2007; Hosaka et al., 2008). The sparseness of the network dictates the shortest path through that network, and hence the length of the resulting chain. An alternative approach uses so-called pruning rules to limit the number of possible connections formed, for example, eliminating all weak synapses (Iglesias and Villa, 2008) or limiting the number of strong synapses (Jun and Jin, 2007). The level of pruning thus determines the width of the chain. Finally, heterosynaptic plasticity can be employed to facilitate chain formation (Doursat and Bienenstock, 2006; Fiete et al., 2010). There a cap was set on the combined weight of efferent and afferent synapses. Including such a limit on pre- as well as post-synaptic weights allows multiple chains to be embedded within one network.

While pruning, heterosynaptic plasticity and other topological constraints clearly play important roles in development, it is important to understand the relative contribution of the STDP rule as distinct from the additional constraints or mechanisms that are used to grow the chain. In fact, it is easy to see that with strict capping conditions in place to limit the size of any layer within the chain, the development of stable synfire chains could be achieved even by a completely random process, in which arbitrary neurons are recruited to the chain. When strong topological constraints are included, one may therefore ask to what extent the details of the learning rule are important at all.

Here, we ask whether it is possible to grow synfire chains in any other way (excluding any form of capping rules). If so, we ask what forms of STDP rule may be suitable, and what is the role of synaptic plasticity in the growth process. Of particular interest to us is a triphasic STDP rule that contains a second depressive region for large positive time differences. Nishiyama et al. (2000) found such a depressive region for time differences greater than 15 ms in rat hippocampal slices. Wittenberg and Wang (2006) also used rat hippocampal slices, and found a depressive region at time differences greater than 25 ms.

In this paper we begin by describing the collapse of synfire chains that are subject to the classical STDP rule in the absence of topological constraints. We argue that triphasic STDP rules can maintain stability in existing chains, even in the absence of any topological constraints. We go on to develop a model based on triphasic STDP. We show that a model incorporating triphasic STDP and an additional non-topological constraint (activity-dependent excitability) leads to the growth of stable chains that scale with network size. The emerging chains are characterized by a range of layer widths and chain lengths. Finally, we show that a triphasic rule can be used to develop multiple chains within a network.

In our model, as in others (Doursat and Bienenstock, 2006; Jun and Jin, 2007; Liu and Buonomano, 2009; Fiete et al., 2010), the presence of spontaneous activity is crucial to the growth of synfire chains. Long before synapses become active in neural tissue, neurons are believed to display spontaneous, sparse spiking activity (Feller, 1999; O'Donovan, 1999; Weliky and Katz, 1999; Blankenship and Feller, 2010) and such activity plays a critical role in development (Syed et al., 2004; Moody and Bosma, 2005; Warland et al., 2006; Tritsch and Bergles, 2010). However, spontaneous activity has been observed to diminish once neurons are part of an active network (Syed et al., 2004; Moody and Bosma, 2005; Warland et al., 2006; Tritsch and Bergles, 2010). Here, we take inspiration from these observed features of developing neural tissue: a key role for spontaneous activity and its suppression in more mature, functional networks. Specifically, in our model, neuronal excitability is taken to be activity dependent, so that spontaneous activity is suppressed among neurons within active chains. This feature leads to stable and strictly feed-forward synfire chains.

## 2. Results

### 2.1. Classical STDP leads to chain collapse

Consider a very small synfire chain, only three layers, each containing one neuron. The first neuron, the input, initiates the chain. With each input spike, the activity propagates down the chain with fixed propagation delays; the input triggers activation of the first layer, then the second. Now consider that the synapses in this fully connected network are plastic and evolve in line with the classical (Bi and Poo, 1998) STDP function (Figure 1D). With every instantiation of the input, the synapse between the input and the first layer will be potentiated, and similarly the synapse between the first and second layer. Fatally for the chain structure, as the time difference between input and layer two is positive, it also falls within the positive tail of the exponential curve and this synapse will also be potentiated. After sufficiently many repetitions of the input, the potentiation of this long range projection from the input to layer two will cause the second layer to fire directly after the input. Hence, the chain collapses (see Figures 1A–C for a schematic illustration). This small example applies generally to any size (length and width) of network, and outlines why, in networks with sufficiently dense initial connectivity, classical STDP alone is not sufficient for the development of synfire chains, or even for maintaining the stability of existing chains.

**Figure 2:**
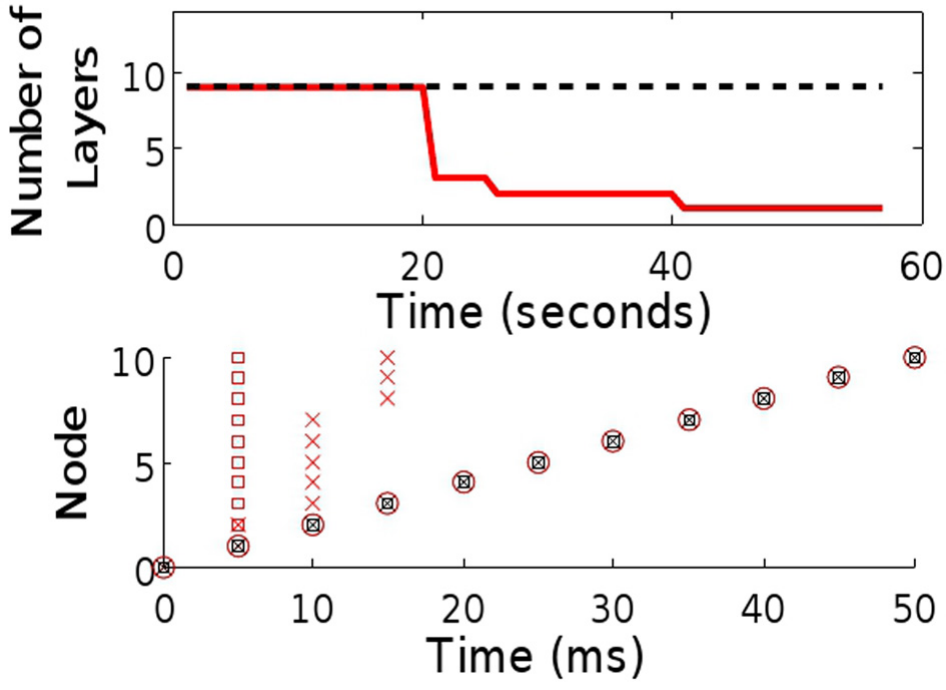
**Comparison of chain maintenance with classical and triphasic STDP. Top:** The number of layers in a chain structure where synapses are allowed to evolve in line with classical STDP (solid, red) and triphasic STDP (dashed). **Bottom:** Sample raster plots of both systems at the beginning (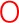), after 25 s (×) and after 50 s of simulated time (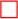).

To illustrate this argument we simulated a small (10 neuron and initially 10-layer) synfire chain embedded within a fully recurrent network. The neurons are binary (see “Methods”) and weights between them set to 0 except for those that form the chain, (*w*_*i i* + 1_), which are set to the firing threshold. The resulting spatiotemporal pattern, a string of single spikes (Figure 2), is a result of the simple (static) chain. As the STDP rule begins to shape the network, the activity pattern changes, with increasing numbers of neurons spiking synchronously due to the reduction in the number of layers in the chain. Before long, the entire chain has collapsed: the activity pattern consists of a synchronous set of network spikes following each input spike.

It is clear that the cause of the destabilization is the long exponential tail in the classical STDP rule. A rule without this long tail, or with a depressive regime at large positive spike-timing-differences may not lead to collapse. We suggest that a triphasic rule as reported in Nishiyama et al. (2000) and Wittenberg and Wang (2006) may be employed for this purpose. Taking inspiration from these studies, we define a triphasic STDP rule

(1) $$\Delta w_{ij}=A\text{​}\left[1-\frac{{\left(\Delta t_{ij}-\alpha \right)}^{2}}{\alpha^{2}}\right]\exp\text{​}\left(\frac{-|\Delta t_{ij}-\alpha |}{\alpha }\right)\text{​},$$

where Δ*w*_*ij*_ is the change in synaptic strength between pre- and postsynaptic neurons *i* and *j*, Δ*t*_*ij*_ is the time difference between spikes in neurons *i* and *j*, α is a scaling parameter that determines the width of the potentiation window (τ_*p*+_ = 2 α) and *A* is the learning rate (see Figure 1E).

Critical to the success of the triphasic rule is the interplay between τ_*p*+_ (the crossing point from potentiation to depression for positive time differences, see Figure 1E) and the time delay *d* associated with spike propagation between two neurons. Specifically, single delays must result in potentiation while multiple delays must depress, such that τ_*p*+_/2 < *d* < τ_*p*+_. Given this STDP rule, we again embed a small chain within a fully connected network. As before, the spatiotemporal string is observed (Figure 2), but now the stability of the structure is maintained.

### 2.2. Growing synfire chains within networks

The simple reasoning above illustrates how triphasic STDP is able to maintain existing chain structures. But, as previously stated, our goal is to demonstrate that a triphasic rule can be used to grow synfire chains within a homogeneous network. Here we use a triphasic rule to grow synfire chains from fully connected networks. Additionally, we show how this process can be simply understood as a bounded random walk.

#### 2.2.1. The model

**Figure 3:**
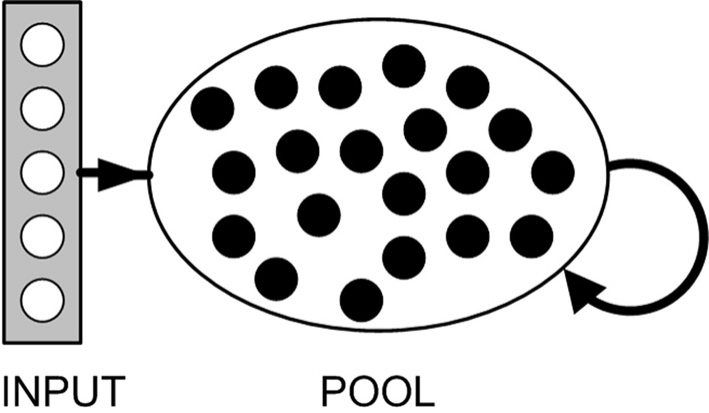
**Network structure.** Arrows indicate full connectivity: Each pool neuron has connections from every input neuron and is reciprocally connected with every other neuron in the pool. All connections are excitatory. Pool neurons fire spontaneously at a low rate. Input neurons fire synchronously at a considerably higher rate.

Model equations are given below (see “Methods”). Briefly, our model consists of a pool of binary, excitatory neurons, each associated with a firing threshold θ. A small set of input neurons spike synchronously at fixed intervals (multiple sets of input neurons are considered later). Input neurons project excitatory connections onto all pool neurons (Figure 3). Synapses are all plastic and associated with a fixed time delay. Spontaneous activity in any pool neuron results in STDP, both in afferent synapses from the input neurons, and in synapses with other pool neurons. A sufficiently low rate of spontaneous activity (relative to the input frequency) ensures that plasticity between pool neurons does not disrupt the chain growth. With an appropriate STDP rule, the above description is sufficient to grow some synfire chains, but does not, on its own, guarantee chain stability.

To achieve stability in developing or developed synfire chains, our neurons display activity-dependent excitability which results in a suppression of spontaneous activity as soon as a neuron is recruited to the chain. This ensures that the chain remains strictly feed-forward, eliminating any scenarios in which an already recruited neuron spikes spontaneously during activation of the chain but before or after its rank in the chain. Had such spontaneous events been permitted, the STDP rule would promote the incorporation of the spontaneously active neuron into the (new) corresponding layer within the chain, which may result in feedback projections and hence cyclic activity patterns. Such loops disrupt chain formation and can, in some cases, cause the collapse of existing chains.

As mentioned above, the key components of the STDP function in our model are potentiation for small positive spike time differences; depression for longer positive time differences, with a crossover to depression between one and two time delays (e.g., two layers apart in an existing chain); and depression for negative time differences. This is implemented in our model with a modified Mexican-hat function [Equation (4) in the “Methods”]. The width of the depressing regimes in this function determines how many layers (forwards and backwards) within a chain will be depressed. The parameters are tuned so that maximal potentiation (STDP peak) is significantly stronger than maximal depression (two STDP troughs), but the time window corresponding to potentiation is significantly narrower, leading to occasional strong potentiation and more frequent, typically weaker depression. By additionally including a very small and constant depression step for very long spike time-differences (both positive and negative), STDP due to effectively uncorrelated spontaneous activity is very slowly depressed. This small modification of the STDP function is sufficient to ensure scalability to any size network (or any number of layers in a chain). The same effect could have been achieved by weak synaptic fatigue rather than directly via the STDP function, but the modification of the STDP function is convenient.

#### 2.2.2. Synfire chain development

Initially, all synaptic weights are set to 0 such that all the activity in the network at this stage is spontaneous (except that of input neurons). The development of synfire chains in our model is driven by spontaneous events in pool neurons that occur within relatively short time intervals before or after a set of input events. A spontaneous spike in a pool neuron directly after an input neuron spike leads to potentiation. For perfectly synchronized inputs, each spontaneous event in a pool neuron will result in identical potentiation or depression of connections from each of the input neurons onto that pool neuron. An accumulation of potentiation events at these connections, with sufficiently low intervening depression, will result in suprathreshold activation of that neuron by the inputs and its recruitment onto the first layer of the chain. Once recruited, a neuron will spike directly after every presentation of the input. Thus, the relative rates of potentiation and depression determine the rate of recruitment of neurons from the pool onto the first layer.

Similarly, neurons in the first layer spike at the same rate as the input, and therefore, are able to recruit a second layer. This process is repeated again by each additional layer. Each new layer consists of perfectly synchronized neurons, resulting in identical potentiation or depression events between each member of the layer onto any particular pool neuron. Note that the perfect synchrony imposed in our model is merely a consequence of its simplicity (namely, the instantaneous, memoryless dynamics of the binary neurons) and does not constitute a general requirement.

**Figure 4:**
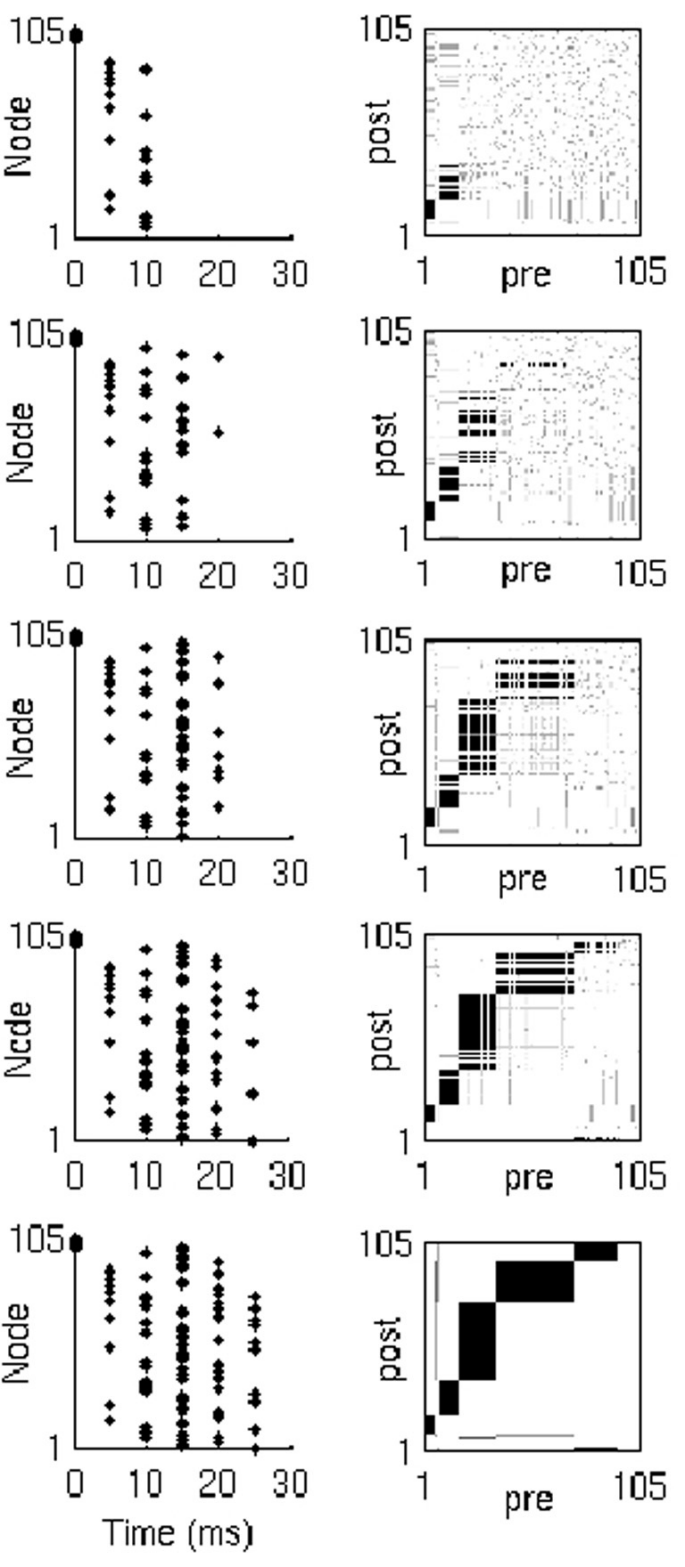
**Network activity and synaptic weights.** Network activity **(left)** and weight matrix evolution **(right)** during synfire chain development (from top to bottom). Neuron indices in weight matrices have been sorted by spike time order (in response to the last input in the simulation), and thus represent the neuron's position in the developed chain.

Layer formation is driven entirely by the form of the triphasic STDP rule. As soon as the first layer begins to form, it begins to recruit the second layer. However, the synapses from layers with few neurons must reach higher weights to induce spikes in (and consequently recruit) pool neurons. Therefore, recruitment onto the second layer will accelerate as the first layer grows, and so on for further layers. Note that with no hard limit on the number of neurons in a layer or on the number of the efferent or afferent synapses of a neuron, recruitment continues to all layers throughout the simulation. In other words, layers are added to the end of the network, whilst at the same time, existing layers continue to grow in size, until all neurons are recruited onto the chain (Figure 4).

**Figure 5:**
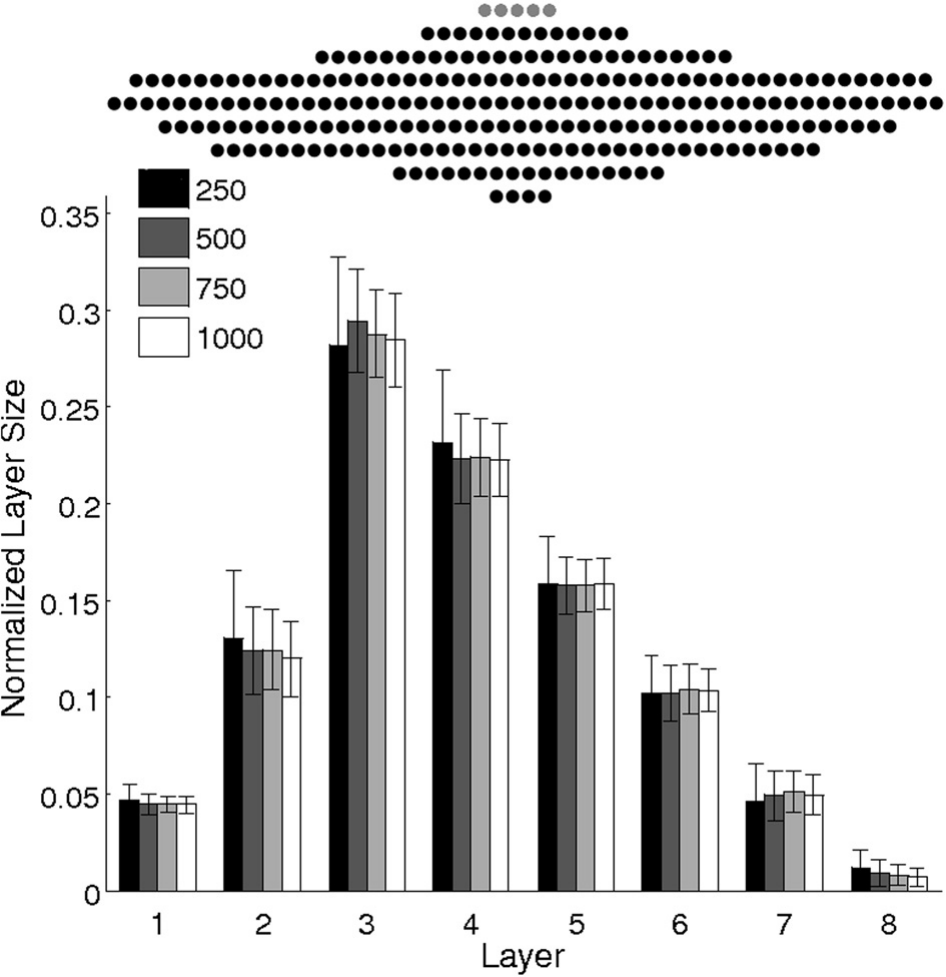
**Scaling of network size: Mean layer sizes ± standard deviations for different size networks (100 runs).** Layer sizes are normalized by the number of pool neurons (see legend). The number of input neurons and learning rate are scaled accordingly. The chain structure follows a consistent pattern that scales with network size, preserving the number of layers and their relative sizes. **Top:** A sample network of 250 pool neurons (black) and five inputs (gray).

Importantly, the rate of recruitment depends on the current state of the network. In particular, the variable layer sizes and the competition among layers to recruit result in dynamic modulation of each layer's recruitment rates. From the perspective of the pool neuron, its afferent connections undergo gradual potentiation from each possible recruiting layer of the chain until one layer wins, at the moment of recruitment; at this point, the repeated activation of the neuron leads to rapid depression of all competing connections from the non-recruiting layers. Eventually, all neurons are depleted from the pool onto the maturing chain. The progressive modulation of each layer's recruitment rate leads to a characteristic chain structure, as shown in Figure 5 for a variety of network sizes.

In fact, the numerical evidence suggests that the model presented here is scale-invariant, so arbitrarily large networks could be used to grow synfire chains whose layer sizes (upon normalization by network size) follow a universal function. Figure 5 suggests that increasing the network size maintains the number of layers while increasing layer widths such that the relative profile (given by the relative widths of the layers) is conserved. Note that to achieve this scale invariance, the width of the input layer is also scaled. As we will see later, the width of the input layer plays a similar role to the learning rate in modulating the number of layers and their width profiles.

To better understand this recruitment process and the factors shaping and modulating the topology of the chain, it is helpful to describe the growth process in terms of a bounded random walk.

### 2.3. Random walk

Momentarily disregard the neurons in our model and consider only the fluctuating synaptic weights. Because the magnitude and rate of these fluctuations are determined by our simulation parameters: the learning rate and the spontaneous and input firing rates, it is possible to model their trajectory before recruitment as a random walk. For the first recruitment to the chain this problem is addressed analytically giving the expected first recruitment time. For recruitments to subsequent layers, the problem is slightly more complex, and thus we simulate the random walk numerically.

**Figure 6:**
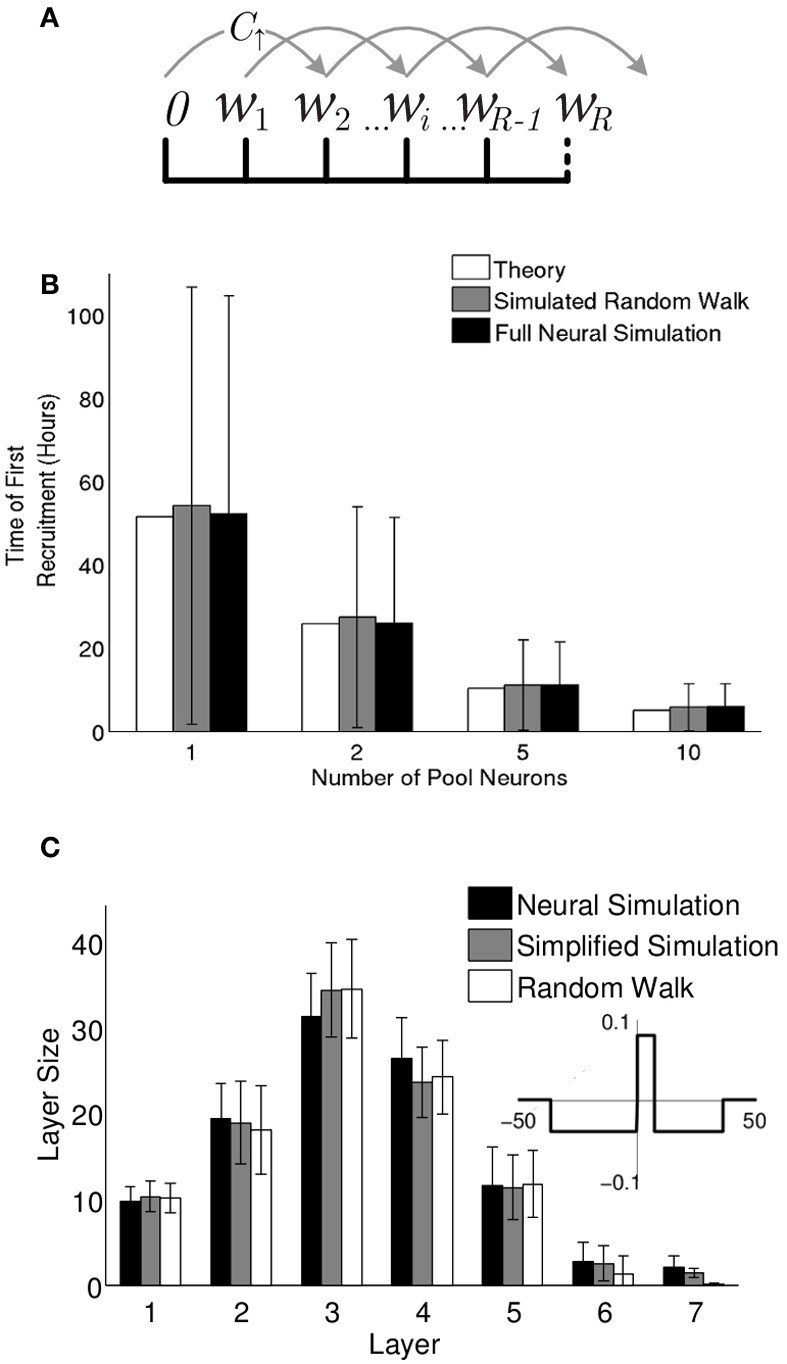
**Synfire chain growth can be described as a random walk. (A)** When the step rule is used, the synaptic weights fall into one of a set of discrete bins. Synaptic weights hop among the bins with probability per unit time (C_↑_) and (C_↓_) (see “Methods”). If a weight reaches the final bin, (*w*_*R*_), the neuron is recruited and the weight exits the random walk. **(B)** The expected time of the first recruitment as calculated (see “Methods”) compared to the mean and standard deviation of first recruitment times in random walk simulations (10,000 runs) and full neural simulations (1000 runs). **(C)** Multi-layer chain structures that evolve from the full neural simulations (black) vs. simplified neural simulations (gray) and random walk simulations (white). Mean and standard deviation of layer sizes were calculated over 500 runs. The simplified neural simulations used the “step rule” (inset) with delay (d = 7.4 ms and each pool neuron's efferent synaptic weights reset to 0 at the time of its recruitment onto the chain.

For tractability, consider a simplification of our triphasic STDP function to a piecewise constant function (henceforth dubbed a “step rule”) given by Equation (5) and schematically illustrated in the inset of Figure 6C. This function approximately replicates the overall ratios of potentiation and depression from the triphasic rule (Equation 1). As in the triphasic rule, depression occurs over a much wider range of time delays, but with lower magnitude, and potentiation is limited to positive spike time differences that are less than two transmission delays.

#### 2.3.1. First recruitment time

Let us concentrate on the very first recruitment event, and specifically on the expected first recruitment time. The system consists of *N* pool neurons and *N*_in_ input neurons, with all initial weights set to zero. Pool neurons display Poisson firing at a rate λ_*p*_, and input neurons fire regularly at a rate λ_in_. For the step rule, the probability per stimulus for the potentiation or depression of a given synapse is constant and given by the probability of coincident input and pool spikes within the corresponding (potentiation or depression) window of the STDP function. The probability of depression is larger, simply because of the larger depression window, and is limited to positive synaptic weights (see “Methods”).

We denote the discrete potentiation and depression steps of the step rule *A*_*p*_ and *A*_*d*_. Without loss of generality, we take the potentiation step *A*_*p*_ to be double the depression step *A*_*d*_. Thus, the possible set of weights a synapse can have is discrete (Figure 6A) and defined as

(2) $$w_{ij}\;\epsilon \;\{kA_{d},k=0,1,\ldots ,R\}.$$

Thus, up until recruitment time, a pool neuron's synaptic weights hop among each of the *k* bins in a (biased) random walk, with the requirement for positive (excitatory weights) represented as a reflecting boundary condition at *k* = 0. Since each neuron sees identical input spikes, the input weights to any given neuron move in tandem. Initially input spikes will not cause postsynaptic firing. Only when the combined input reaches the firing threshold *N*_in_ *w*_*ij*_ ≥ θ will a postsynaptic spike be generated, immediately recruiting this neuron to the first layer. At this point, the spontaneous activity for this neuron is stopped, which prevents further movement of its afferent weights and ensures that once a neuron is recruited to a layer, it cannot be recruited elsewhere. Thus, this weight leaves the weight pool and its random walk is terminated. (Mathematically this is represented by an absorbing boundary condition at *R* = ⌈ θ/(*N*_in_*A*_*d*_)⌉, so *w*_*R*_ ≥ θ/*N*_in_).

The formulation of the dynamics of pool weights in terms of a random walk allows us to calculate analytically the expected time of the first recruitment for any given parameter set (see “Methods”). In Figure 6B these results are compared to our numerical random walk (with discrete inputs) and the full neuronal simulations and show agreement across all three methods. Thus, the recruitment process into the first layer is accurately described by a bounded random walk, as demonstrated here for the first recruitment. For later recruitments onto the first layer and prior to the formation of a second layer, the only correction needed is that the pool of available neurons is gradually depleted.

#### 2.3.2. Subsequent layer formation

When describing the development of the entire chain, we think now not of one random walk but of multiple concurrent random walks. If plasticity events were independent, these random walks would exactly describe chain development in our system. In fact, the spike-timing-dependence in the learning rule introduces some correlations in plasticity events. In what follows, we first describe the random walk formulation and further approximations that simplify our model, and then compare this model with neural simulations.

Consider three classes of random walks. Firstly, as before, the recruitment of the initial layer is driven by plasticity events between the pool neurons and the inputs. Secondly, there is also plasticity between the individual pool neurons. Intra-pool interactions occur with much lower probabilities (due to the low spontaneous firing rates). Nonetheless at the time of recruitment onto the chain, a pool neuron may already have some non-zero weights in its connections with the remainder of the pool. To neglect the effect of the second set of (intra-pool) random walks, in our simulations, we reset efferent weights of newly recruited neurons to 0. (Simulations including intra-pool dynamics give quantitatively similar results, indicating that the contribution of intra-pool dynamics is very small, data not shown). Finally, during recruitment to subsequent layers of the chain, each pool neuron undergoes random walks that compete to recruit it onto each of the existing layers in the chain.

For any layer ℓ of size *N*_ℓ_, recruitment onto the subsequent layer occurs when the combined efferent weights onto a pool neuron *j* reach the threshold, $w_{j}=\sum_{i\;=\;1}^{N_{\ell }}w_{ij}\geq \theta$. Thus, for every pool neuron, the combined afferent weight *w*_ℓ*j*_ undergoes a random walk with dynamic step sizes for potentiation N_ℓ_ · *A*_*p*_ and depression *N*_ℓ_ · *A*_*d*_. Note that for sufficiently large layers (with *N*_ℓ_ ≥ θ/*A*_*d*_), a single set of potentiation events (following a single spontaneous spike) is sufficient for recruitment. It is this dependence of layer size on the rate of recruitment that gives our networks their characteristic shape. With the input layer size fixed, neurons are initially recruited onto layer one with constant rate. When recruitment into layer two begins, the recruitment rate onto layer one begins to diminish due to the competition. At some point, *N*_1_ will outnumber the number of the inputs, at which time recruitment onto the second layer will be faster than recruitment onto the first layer. Thus the second layer will soon outnumber the first, and so on, until the maximum growth rate is reached.

To compare the above random walk model with neural simulations, a number of simplifications are implemented in the neural simulations. These include the elimination of intra-pool dynamics, a small adjustment of neuronal delays, and the elimination of plasticity for large (positive or negative) spike-time differences (see “Methods”). With those simplifications, the growth process and final network structures predicted by the random walk model neatly match the simplified simulations (Figure 6C). The near match even to the full neural simulations shows that for the parameter regimes described here, the recruitment dynamics is well described by a set of independent recruitment events. Furthermore, the tractability of the random walk description allows insight into the recruitment process that may not be immediately evident from the simulations, most notably, the relationship between layer size and recruitment rate. Understanding these dynamics allows us to return to the full simulations and modulate the network structure.

### 2.4. Modulation of network structure

**Figure 7:**
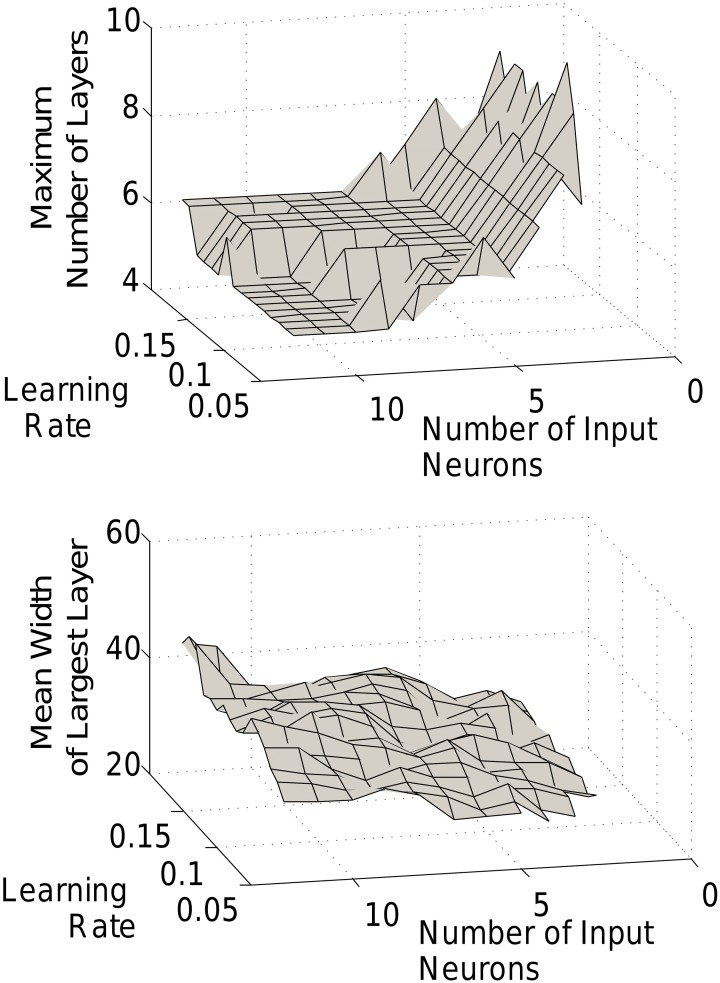
**Effect of learning rate and input size.** The length of the final chain **(top)** and the width of the largest layer **(bottom)** are dependent on the learning rate and input size.

In Figure 5 we saw the characteristic shape of the networks produced by our triphasic learning rule for different size networks. In fact, the general features of the chain development and structure are robust to changes in a wider range of parameters. Grossly, chains will form with relatively narrow early layers, progressively wider middle layers, and decreasing layer sizes toward the end of the chain. That said, the detailed chain structure (the mean number of layers and the relative layer sizes) can be modulated by the number of input neurons or the learning rate (Figure 7).

Increasing the number of input neurons produces a shorter, fatter network, i.e., with larger but fewer layers. Smaller input layers lead to longer thinner chains. Indeed, as mentioned above, recruitment rates, and hence layer sizes depend strongly on the size of the recruiting layer. With a small input group and therefore a slow recruitment rate, the subsequent (larger) layers will quickly become the dominant recruiters, thus rapidly increasing the number of layers. Conversely, with a large input, recruitment remains closer to the head of the network for much longer. The amplitude of the STDP function (the learning rate), can similarly modulate the length to width ratio of the network. With lower learning rates, the recruitment is slower (just as with smaller input layers), leading to slower development and longer and thinner chains.

### 2.5. Multiple inputs

In the results described so far, there has been one distinct set of inputs projecting onto the remainder of the network. However, in the brain, there are often multiple inputs which compete with each other for control of target neurons. For example, the projections from either eye segregate the target tissue into ocular dominance columns. The development of synfire chains with multiple inputs or the embedding of multiple chains within a network have been topics of recent interest (Aviel et al., 2003; Teramae and Fukai, 2007; Liu and Buonomano, 2009; Fiete et al., 2010). In this section we ask what happens if our system includes multiple inputs. Furthermore, how does the number of distinct inputs affect the final network configuration?

**Figure 8:**
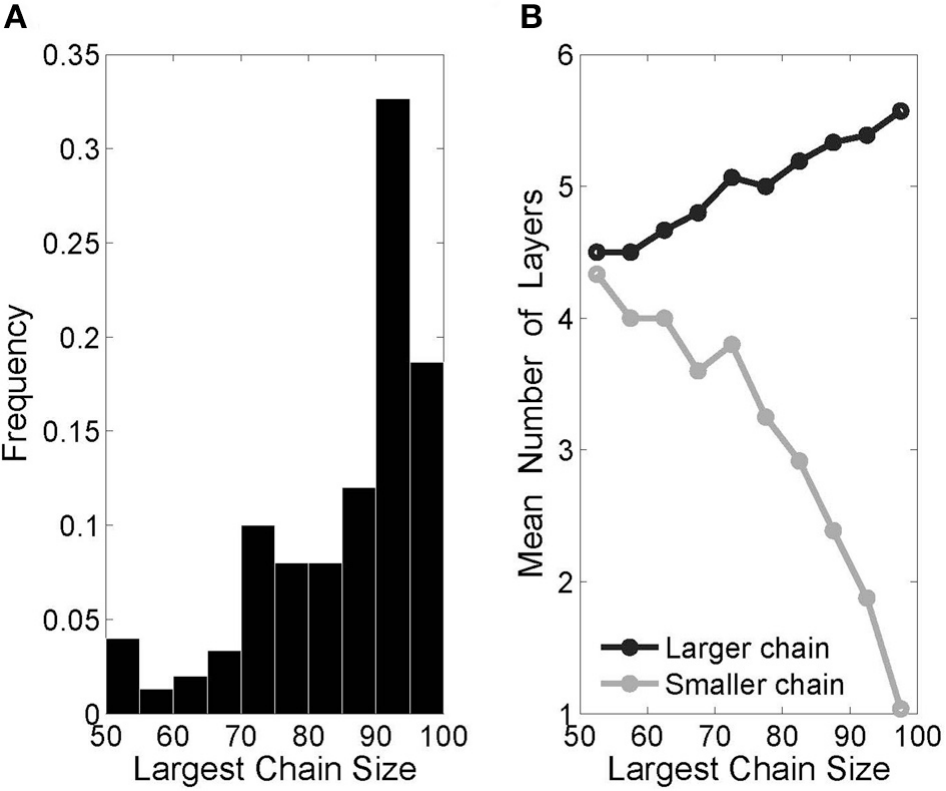
**Two-chain networks with two inputs. (A)** Histogram of “largest chain size,” defined as the number of neurons in the larger of the two chains (the small chain size is the complement to 100—the total number of pool neurons), from 150 runs. **(B)** The difference in mean lengths between the larger and smaller chains is in keeping with the divergence in chain size.

In agreement with previous authors (Liu and Buonomano, 2009; Fiete et al., 2010), we have found that multiple inputs lead to the development of multiple synfire chains within one originally homogeneous network. Adding a second input group to our original setup (Figure 8) resulted in two distinct chains. Importantly, there usually emerged one dominant chain that grew larger and subsequently faster than the other. This phenomenon can be directly attributed to the described effect layer size has on recruitment rates. The initial symmetry breaking between the two chains occurs by chance, but immediately leads to a positive feedback loop, which only intensifies as the chain sizes diverge.

There are various plausible instances for which similar competition between inputs may be beneficial, e.g., at the boundaries between maps (frequency maps in primary auditory cortex or whisker response maps in barrel cortex). However, when balanced representation of inputs is important, the dynamics observed here are likely to be detrimental. The possibility of mitigating these effects through balancing inhibitory networks [e.g., similarly to Dorrn et al. (2010)] therefore raises interesting questions.

Increasing the number of inputs beyond two only intensifies the symmetry breaking described above. Simulations with up to ten input groups show that the majority of networks display one long chain and a number of smaller chains. Here too, most chains tend not to overlap (data not shown).

### 2.6. Full neural simulations: leaky integrate and fire model

Our results using binary neurons show how triphasic STDP can be used to grow synfire chains without additional topological constraints. Nonetheless, this approach was only tested in relatively small networks of binary neurons. To test this plasticity rule with more biologically realistic neural models, network sizes and connectivity, we implemented triphasic STDP in a network of 10,000 leaky integrate and fire (LIF) neurons with sparse (6%) random connectivity (see “Methods”).

**Figure 9:**
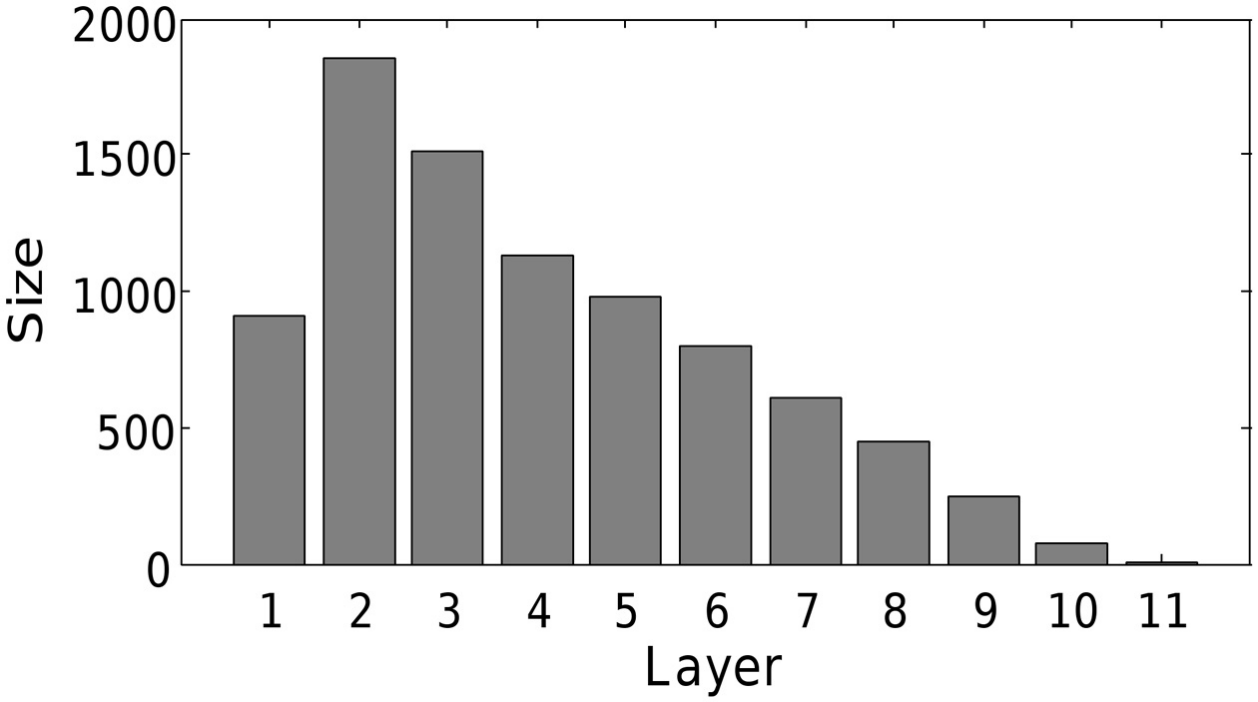
**Structure of network with 10,000 neurons.** All other parameters as in Table 1. Note that only 8551 of the neurons were recruited due to limits on computational time.

We find that the mechanism scales to realistic network sizes. At the end of the simulation, 8551 of the 10,000 neurons were recruited into a single chain of 11 layers. This final network structure is presented in Figure 9. Hence, we conclude that the combination of triphasic STDP and activity-dependent excitability is also sufficient to grow synfire chains in more biologically realistic networks. Note that due to the reduced connectivity, there is no longer all-to-all feed-forward connectivity between contiguous layers. The reduced connectivity increased the number of layers, and slightly modified the network structure, but grossly maintained a qualitatively similar diamond like shape. The increase in neuron numbers and reduction in connectivity should further increase the possibility of embedding multiple chains. To do this, one could include multiple input groups as above.

## 3. Discussion

Here we have presented a model for the development of synfire chains that relies on a triphasic STDP rule and activity-dependent excitability. Within this model, there is no need to apply any topological constraints either on the initial connectivity or the evolving connectivity in the system. Indeed, we demonstrate that synfire chains can grow from initially homogeneous, fully connected networks. The development of synfire chains without a limit on synaptic connections is a novel contribution to this topic of long-standing interest in computational neuroscience (Abeles, 1982, 1991; Bienenstock, 1991, 1992; Diesmann et al., 1999; Gewaltig et al., 2001; van Rossum et al., 2002; Aviel et al., 2003, 2005; Ikeda, 2003; Kitano et al., 2003; Tetzlaff et al., 2004; Doursat and Bienenstock, 2006; Ishibashi et al., 2006; Jun and Jin, 2007; Masuda and Kori, 2007; Hosaka et al., 2008; Iglesias and Villa, 2008; Kumar et al., 2008; Liu and Buonomano, 2009; Fiete et al., 2010; Shinozaki et al., 2010). In fact, as recently shown (Kunkel et al., 2011), classical STDP [as in Bi and Poo (1998)], without additional limitations on connectivity, is insufficient for chain development.

STDP has been found to occur within the song production system of zebra finch (Boettiger and Doupe, 2001). However, the form of any STDP function within HVC (the site of postulated synfire chains) has yet to be determined. The triphasic STDP functions proposed here offer an interesting alternative for growing synfire chains and, we believe, a new and exciting direction for experimental research in songbird HVC and beyond. Interestingly, in our model, chain growth follows a self-organization process that leads to chain profiles that scale with network size and are not dissimilar in gross features to the layers of the cortex (Landing et al., 2002). Modulations in the number of input neurons and plasticity parameters can be used to fine tune the structure of the chain. Our results suggest that in areas of the brain where pruning does not dominate development, the detailed activity-dependent form of the local neuronal and synaptic plasticity rules may play an important role in shaping the developing pattern. In the context of songbird HVC, the network structures obtained (with 6–10 layers) and network activation pattern [*O*(100) ms in duration] would be consistent with the postulated encoding for the sequences of notes making up a syllable (or basic unit) of the bird song.

The growth and stability of our synfire chains rely on a class of STDP functions that are characterized by (1) potentiation for short pre- to postsynaptic spike time differences, corresponding to connections that project only one layer forward and (2) depression for longer spike time differences, corresponding to connections that project two or more layers forward, and for negative time differences, for backward-projecting connections. A wide class of STDP functions with this general form can successfully be used to develop synfire chains. One example may be a modified two-exponential STDP function in which the positive-time exponential is offset to become depressive for longer time intervals, longer than a single propagation delay (Delgado et al., 2010; Waddington, 2011). We conclude that with any variant of the triphasic rule, a stable feed-forward chain will successfully evolve conditioned on (1) the choice of time scales (and overall probability to potentiate or depress); (2) an appropriate balance of spontaneous activity and input rate; and (3) fixed propagation delays. These conditions are now discussed in turn.

Within this class of STDP functions, the relative strength of potentiation to depression (and their associated probabilities) in the STDP function determines the possibility, and if so speed of layer formation and can be used to modulate the emerging chain structure. The requirement for a strictly feed-forward chain (or absence of loops) implies that distant connections across the network (particularly backward projecting ones) must remain weak over time scales of development. For sufficiently large networks (or numbers of layers) to support stable chain structures, we have incorporated a very weak (almost negligible) depressive regime in our STDP function. With this additional feature, the scaling of synfire chains with network size is ensured.

In our model, stochastic spontaneous activity is critical to the development of chains, but stops immediately upon recruitment. This suppression of spontaneous activity is qualitatively consistent with many reports in the literature (Syed et al., 2004; Moody and Bosma, 2005; Warland et al., 2006; Tritsch and Bergles, 2010), although it is likely that *in vivo* the transition would not be as sharp. Specifically, our model suggests that in regions displaying precise spatiotemporal activity patterns, and where the STDP rule plays a major role in development, suppression of spontaneous activity may coincide with recruitment of neurons onto these patterns. With suppression of spontaneous activity in place, the above triphasic STDP function indeed leads to stable and strictly feed-forward structures. However, *in vivo*, it is certainly plausible that synfire chains co-existwith underlying recurrent microcircuitry (i.e., containing loops). Indeed, if synfire chains are used in working memory and other pattern maintenance systems, such loops may be required.

We should also note that in our model the time difference used to calculate STDP and the transmission delay are the same. Classically the time difference used to measure STDP is taken from the post-synaptic potential arrival at the dendrite to neuron firing. In our leaky-integrate-and-fire simulations, we have set our transmission delay at 5 ms and also use this as the time difference for the STDP calculation. This assumes that the axonal delay is roughly equal to the dendritic delay, so that the time between spikes is the same as the time taken for the EPSP to reach the soma and then back-propagate. It further assumes that propagation delays dominate over the synaptic delay which is neglected. For networks in which this assumption breaks down and synaptic time delays dominate, these time delays may themselves be modulated by the plasticity rule (with higher efficacies having faster synaptic transmission)—as scenario not considered in our model. Furthermore, in reality, delays are heterogeneous and if our STDP rule were to contribute to network formation, one may expect the observed values of τ_*p*+_ to vary as well. As there are only few reports of triphasic STDP in the literature to date (Nishiyama et al., 2000; Wittenberg and Wang, 2006), it is too early to tell whether changes in the potentiation window of the STDP function correlates in any way with transmission delays in the corresponding systems.

To better understand the self-assembly of the circuit, we formulated the recruitment process in terms of a random walk that is then amenable to population density analysis. To our knowledge, this is the first study that uses a population density of weights, rather than one of neurons (which have been used routinely). The parameters in our model give rise to an interesting dynamical regime, in which depression dominates most of the time. Viewed as a continuous-time random walk, the recruitment process can be described by rare upward hopping events, that are typically overshadowed by a strong downward drift. Recruitment of neurons onto the chain is therefore a rare and relatively rapid series of events. The ability of the same class of STDP rules to drive chain-formation outside of this parameter regime is an interesting and open question.

The link between plasticity of individual synapses or neurons and network properties is a fundamental and exciting problem in neuroscience, if often an elusive one. In the context of synfire chains, pruning and other capping rules that limit numbers of synapses offer a mesoscopic solution to the self-assembly problem. In contrast, the triphasic STDP rule, complemented by activity-dependent plasticity provides a different solution, in which the emerging structure is shaped by the interplay between local (synaptic) and network-wide dynamics. Specifically, the chain profile is determined by global competition between the different layers (i.e., by the functional circuit) over recruited connections. While it is clear that development in neural tissue relies on the complex and delicate balance of many mechanisms on a variety of time scales, our results suggest that plasticity rules based on spike timing could potentially play a very important role in molding the functional circuit structures in the nervous system.

## 4. Methods

### 4.1. Binary neural simulations

We use binary neuronal states *S*_*j*_ ϵ {0, 1} and continuous-valued, instantaneous membrane potential *V*_*j*_ (i.e., infinite leak) to determine neuronal spiking (*S*_*j*_ = 1). The potential at every point in time (*t*) sums over contributions from other spikes occurring precisely one time-delay earlier *t* − *d* and is defined as,

(3) $$\begin{array}{l} V_{j}(t)=\sum_{i\;=\;N_{\text{in}}\;+\;1}^{N\;+\;N_{\text{in}}}S_{i}(t-d)w_{ij}\\ S_{i}(t)=\{\begin{array}{cc} 1 & V_{i}\geq \theta \\ 0 & V_{i}<\theta , \end{array} \end{array}$$

where *w*_*ij*_ is the weight of the synapse from any neuron *i* to a pool neuron *j* and θ is the firing threshold. Once a spike has occurred a neuron enters an absolute refractory period *t*_ref_. The time delay *d* denotes the spike-to-spike transmission time and is assumed to be equal to the Δ*t* argument in the STDP functions (denoting the time difference between the arrival of the pre- and post-synaptic spikes at the synapse). All synaptic weights *w*_*ij*_ are initially set to 0 and evolve according to one of three plasticity functions.

In triphasic STDP,

(4) $$\begin{array}{l} \Delta w_{ij}=\{\begin{array}{cc} A[1-\frac{{\left(\Delta t_{ij}-\alpha \right)}^{2}}{\alpha^{2}}]\times \exp(\frac{-|\Delta t_{ij}-\alpha |}{\alpha }) & -50\text{ms}\leq \Delta t_{ij}\leq 50\text{ms}\\ \Delta w_{ij}{|}_{\Delta t_{ij}\;=\;50\text{ ms}} & \Delta t_{ij}>50\text{ms}\\ \Delta w_{ij}{|}_{\Delta t_{ij}\;=\;-50\text{ ms}} & \Delta t_{ij}<-50\text{ms}, \end{array} \end{array}$$

In other words, this function is identical to Equation (1) except that Δ*w*_*ij*_ is fixed at its values of Δ*t*_*ij*_ = ±50 ms for longer time intervals |Δ*t*_*ij*_| > 50 ms; this fixing of Δ*w*_*ij*_ gives a small constant depression (< 1.5 × 10^−3^).

In the step STDP function, the potentiation and depression are piecewise constant

(5) $$\Delta w_{ij}=\{\begin{array}{cc} A_{d} & \tau_{d-}<\Delta t_{ij}<0\\ A_{p} & 0\leq \Delta t_{ij}<\tau_{p+}\\ A_{d} & \tau_{p+}\leq \Delta t_{ij}<\tau_{d+}, \end{array}$$

and zero otherwise. Finally, in classical STDP, we choose an anti-symmetric rule

(6) $$\Delta w_{ij}=\{\begin{array}{cc} A_{c}e^{-\beta \Delta t_{ij}} & \Delta t_{ij}>0\\ -A_{c}e^{\;\beta \Delta t_{ij}} & \Delta t_{ij}<0\\ \;0 & \Delta t_{ij}=0, \end{array}$$

where the amplitude *A*_*c*_ and the decay rate β are taken to be equal for both exponentials. For computational efficiency, the value of Δ*t*_*ij*_ was rounded to the nearest millisecond and Δ*w*_*ij*_ was read from a look-up table. This made no qualitative difference to results. Note that the above rules (Equations 4–6) are additionally constrained so that synapses are excitatory at all times and bounded above with 0 ≤ *w*_*ij*_ ≤ *w*_max_. All STDP pairings are nearest neighbor. Experiments with all-to-all pairing yielded qualitatively the same results (not shown). Simulations were run until all neurons had been recruited.

### 4.2. Simplified neural simulations

For the comparison of random walk results with neural simulations (Figure 6C), a number of simplifications are implemented. First, intra-pool dynamics are eliminated (i.e., synaptic plasticity among unrecruited neurons, which may bias the choice of neurons to be recruited). In fact, this has a very small effect on the recruitment dynamics and chain structure. Second, to ensure that each potentiation event applies at most to a single layer, the delay between neurons is slightly increased to *d* = τ_*p*+_ −0.1 ms. This has a minor effect on chain structure. Finally, we use the simpler “step rule” which has strictly zero plasticity for long spike time differences |Δ*t*_*ij*_| > 36 ms (i.e., < τ_*d*−_ or > τ_*d*+_). This has negligible effect on simulations with relatively small networks, but may occasionally induce recurrent connections and hence collapse of the synfire chains for larger simulations (with thousands or more neurons).

Simulations parameters for both full and simplified neural simulations can be found in Table 1.

**Table 1:** **Simulation parameters**. Unless otherwise noted simulation parameters are as follows.

**BINARY NEURONS—NEURAL AND NETWORK PARAMETERS**					
*N*	100	λ_in_ (Hz)	3.0	*w*_max_	0.7
*N*_in_	5	λ_p_ (Hz)	0.1	*d* (ms)	5.0
		*t*_ref_ (ms)	6.0	θ	1.0
**Triphasic STDP**		**Step rule STDP**		**Classical STDP**	
*A*	0.1	*A*_*d*_;*A*_*p*_	0.04;2*A*_*d*_	*A*_*c*_	0.1
α (ms)	4.0	τ_*p*+_; τ_*d*+_;τ_*d*_– (ms)	7.5; 36; −36	β (ms^−1^)	0.05
τ_*p*_ (ms)	2α	*d* (ms)	7.4		
**LEAKY INTEGRATE AND FIRE NETWORKS**					
*N*	10,000	λ_*p*_ (Hz)	0.1	*d* (ms)	5.0
*N*_in_	200	λ_in_ (Hz)	3.0	*w*_max_ (nS)	20.0
*A*	8	τ_syn_ (ms)	0.2	θ (mV)	−50.0
*t*_ref_ (ms)	20.0	*E*_*L*_ (mV)	−85.0	*V*_reset_ (mV)	−80.0
*E*_ex_ (mV)	0	*g*_*L*_ (nS)	1.125	*C*_*m*_ (pF)	22.5

### 4.3. Random walk

The probability per unit time for the potentiation of a given synapse *C*_↑_ is constant and given by

(7) $$C_{↑}\equiv \lambda_{\text{in}}\lambda_{p}\tau_{\;+}\;e^{-\lambda_{p}\tau_{+}},$$

where τ_+_ = τ_*p*+_ is the width of the potentiation window. The above assumes a regular input pattern (hence the constant λ_in_) and a Poisson firing pattern for the spontaneous firing of the pool neurons; accordingly the probability of a spike within a time window τ_+_ is λ_*p*_ exp (−λ_*p*_ τ_+_). We further assume that the Poisson rate is much lower than the input rate; hence it is sufficient to consider a single pair of input and spontaneous events.

For depression a similar formula holds

(8) $$C_{↓}\equiv \{\begin{array}{cc} \lambda_{\text{in}}\lambda_{p}\tau_{\;-}\;e^{-\lambda_{p}\tau_{-}} & w>0\\ 0 & w=0, \end{array}$$

where τ_−_ = τ_*d*−_ + τ_*d*+_ is the combined width of both of the depression windows, and synaptic depression is limited to positive weights (for simplicity, the neuronal indices *ij* have been dropped from *w*_*ij*_).

Since we take *A*_*p*_ to be double *A*_*d*_, the weights are restricted to a state space of discrete bins (0, …, *R*) where recruitment occurs in bin *R* (Figure 6A). For very large *N*, or given a large number of repeats of the experiment, a probability density function $\vec{\Omega }(t)$ can be defined, where Ω_n_ is the fraction of the total number of neurons that occupy bin *N*, i.e., whose weights equal *n A*_*d*_. The elements of $\vec{\Omega }$ are non-negative and sum to a value equal to or less than 1. We can now approximate the stochastic process of first layer formation by a master equation for $\vec{\Omega }(t)$ (Larralde et al., 1992; van Kampen, 1992)

(9) $$\frac{d}{dt}\vec{\Omega }=M\cdot \vec{\Omega },$$

with the matrix *M* given by an asymmetric tridiagonal Toeplitz matrix, summarized by

(10) $$\frac{d\Omega_{n}}{dt}=C_{↑}\Omega_{n\;-\;2}+C_{↓}\Omega_{n\;+\;1}-(C_{↓}+C_{↑})\Omega_{n},\;0<n<R-1,$$

with boundary conditions

(11) $$\begin{array}{l} \frac{d\Omega_{0}}{dt}=C_{↓}\Omega_{1}-C_{↑}\Omega_{0}\\ \frac{d\Omega_{1}}{dt}=C_{↓}\Omega_{2}-(C_{↓}+C_{↑})\Omega_{1}\\ \frac{d\Omega_{R\;-\;1}}{dt}=C_{↑}\Omega_{R\;-\;3}-(C_{↓}+C_{↑})\Omega_{R\;-\;1}. \end{array}$$

For a midrange bin Ω_*n*_ rises by potentiation from bin *n* − 2 at rate *C*_↑_ (giving the C_↑_ subdiagonal in *M*, *A*_*p*_/*A*_*d*_ = 2 rows below the diagonal) and depression from bin *n* + 1 at rate *C*_↓_ (giving the (*C*_↓_) subdiagonal directly above the diagonal in *M*); Ω_*n*_ decreases by either potentiation or depression at a rate *C*_↓_ + *C*_↑_ (diagonal terms in *M*). Due to the reflecting boundary condition at bin *n* = 0, this bin's occupancy is governed only by depression onto it and potentiation from it. The upper absorbing boundary condition at *n* = *R* eliminates depression from bin *R* to bin *R* − 1. Together with initial conditions (full occupancy at Ω_0_),

$$\Omega_{0}(0),\Omega_{1}(0),\ldots ,\Omega_{R}(0)=1,0,\ldots ,0,$$

this system determines $\vec{\Omega }(t)$. Note that the master equation formulation above considers uniform hopping rates over continuous time (and hence neglects the discrete times of the stimulation protocol).

#### 4.3.1. First recruitment time

It is now possible to estimate the formation time and the initial speed of formation of the first layer. We begin with the probability that recruitment hasn't yet happened, which is given by the entire contents of bins 0, …, *R* − 1

(12) $$\sum_{n\;=\;0}^{R\;-\;1}\Omega_{n}(t).$$

The probability of recruitment between time *t* and *t* + *dt* is (van Kampen, 1992)

(13) $$\begin{array}{l} f_{1}(t)=-\frac{d}{dt}\sum_{n\;=\;0}^{R\;-\;1}\Omega_{n}(t)\\ \;=C_{↑}\Omega_{R\;-\;1}(t)+C_{↑}\Omega_{R\;-\;2\;}(t). \end{array}$$

so that *f*_1_(*t*) is the probability density for recruitment, for each (independent) random walk. The expected recruitment time 〈*t*_1_〉 is then given by

(14) $$\langle t_{1}\rangle =\int_{0}^{\infty }tf_{1}(t)dt.$$

In a network with *N* pool neurons, we can now calculate the first recruitment time. To find the probability density for at least one neuron being recruited *f*_*N*_(*t*), we calculate the probability that none have been recruited between time 0 and *t* and subtract this from one

(15) $$f_{N}(t)=1-{\left(1-\int_{o}^{t}f_{1}(t)dt\right)}^{N}=1-{\left(\sum_{n\;=\;0}^{R\;-\;1}\Omega_{n}(t)\right)}^{N}\text{​}.$$

The expected first recruitment time is then given by 〈*t*_*N*_〉,

(16) $$\langle t_{N}\rangle =\int_{0}^{\infty }tf_{N}(t)dt.$$

Solutions to Equation (16) were computed by numerically solving the eigenvalue problem in Equation (9). Interestingly, given our parameter regime, the solution yields one dominant eigenvalue (i.e., one dominant time scale), indicating that the recruitment process onto the first layer can be described as a rapid escape (or multiple very closely spaced potentiation events) from the unrecruited pool. Solving for $\vec{\Omega }$ over time confirms this. Initially all weights are zero: All probability is concentrated in bin 0 [Ω_0_ (0) = 1]. This bin initially depletes rapidly due to potentiation and some bins will have higher occupancy, i.e., Ω_1, …, *R* − 1_ will begin to grow. As these bins are populated, depression comes into play, reducing most weights to 0 again. The net result is that the occupancy in higher bins remains almost stationary at a very low value; while the first bin occupancy Ω_0_ depletes very slowly (due to recruitment).

In a relatively small sized network this means that at any time almost no potentiated weights are visible: recruitment into the first layer takes place if a neuron's weights happen to potentiated rapidly a number of times in relatively close succession. This is indeed what is observed. The advantage of our analysis is that we can estimate the time at which the first layer starts to form directly in terms of the simulation parameters.

#### 4.3.2. Chain formation

For recruitment to further layers, we simulate the random walk explicitly. Since all weights from any layer to any pool neuron undergo plasticity in sync, it suffices to simulate *L* random walks for each pool neuron, where *L* is the (dynamic) number of layers in the system. Each weight *w*_ℓ*j*_ is defined as total efferent weight from the layer ℓ = 0, …, *L* to a neuron, *j*, initially set to 0. The magnitude of the plasticity steps are *A*_*p*_*N*_*ℓ*_ (for potentiation) and *A*_*d*_ *N*_*L*_ (for depression), where *N*ℓ is the size of the *previous* layer (or the input in the first instance). As before, probabilities to potentiate and depress are given by *C*_↑_ and *C*_↓_, respectively. Starting from *N*_0_ = *N*_in_ and *N*_ℓ> 0_ = 0, all random walks are run concurrently. Each recruitment event (with some weight crossing threshold) leads to an increment in the corresponding layer size *N*_ℓ + 1_. All random walks associated with this pool neuron are then halted upon recruitment.

### 4.4. Leaky integrate and fire model

The simulation was built using the neural simulation software NEST (Gewaltig and Diesmann, 2007). As in previous models, the network consists of *N* identical network (i.e., pool) neurons and *N*_in_ input neurons. Here connectivity was set at 6%. Each input was randomly assigned connections to 0.06 *N* network neurons. Similarly, network neurons were connected to 0.06 *N* other network neurons. The input neurons have no incoming connections. Network neurons spike with a spontaneous (Poisson) firing rate λ_*p*_. Input neurons spike regularly with a rate λ_in_. All synaptic connections are excitatory. However, the weak synaptic efficacies (relative to the firing threshold) and sparse spontaneous activity within the unrecruited pool of neurons means that this activity cannot propagate.

The neural model was chosen from those distributed with NEST (“iaf_cond_exp”). The sub-threshold membrane potential *V*_*j*_ of neuron *j* evolves according to

(17) $$C_{m}\frac{dV_{j}}{dt}=-g_{L}(V_{j}-E_{L})+g_{\text{syn},j}(V_{j}-E_{\text{ex}}),$$

where *C*_*m*_ is the membrane capacitance, *E*_*L*_ and *E*_ex_ are the reversal potentials of the leak and excitatory synaptic conductances, respectively, *g*_*L*_ is a constant leak conductance, and *g*_syn_ is a non-negative (and initially zero) synaptic conductance. Because all synapses are excitatory, synaptic conductances increase instantaneously with synaptic inputs and decay exponentially otherwise,

(18) $$\tau_{\text{syn}}\frac{dg_{\text{syn},j}}{dt}=-g_{\text{syn},j}\;+\sum_{i\;=\;1}^{N\;+\;N_{\text{in}}}w_{ij}S_{i}(t-d),$$

where τ_syn_ is the synaptic time constant, 0 ≤ *w*_*ij*_ ≤ *w*_max_ is the bounded weight of the synapse and *S*_*i*_(*t*) ϵ {0, 1} is 1 if the pre-synaptic neuron *i* fired at time *t* and 0 otherwise; *d* is the delay between neurons. (Note that for legibility, the index *j* has been omitted from *g*_syn, *j*_.) If the membrane potential *V*_*j*_ reaches the firing threshold θ, the neuron is said to have spiked and *V*_*j*_ is set to the reset potential *V*_reset_. The neuron then enters an absolute refractory period of *t*_ref_, during which *V*_*j*_ is fixed at *V*_reset_. All synapses were modified according to triphasic STDP (Equation 4). All parameters can be found in Table 1.

### Conflict of interest statement

The authors declare that the research was conducted in the absence of any commercial or financial relationships that could be construed as a potential conflict of interest.

## Footnotes

^1^Note that the precise details of the STDP function, typically consisting of a positive exponential for positive pre-to-post synaptic firing time difference and a negative exponential (typically with a smaller amplitude prefactor and a longer time constant) for negative time differences, are inconsequential for our purposes here.
